# （2-羟乙基）-***β***-环糊精键合表面多孔和全多孔颗粒手性固定相在超临界流体色谱和反相液相色谱中的手性分离性能比较

**DOI:** 10.3724/SP.J.1123.2025.12017

**Published:** 2026-09-08

**Authors:** Guangyong YANG, Haijiang WU, Huangtao ZHANG, Hongli WANG, Qiuyan LIANG

**Affiliations:** 新疆维吾尔自治区质量基础发展研究院，新疆 乌鲁木齐 830011; Research Institute for Basic Quality Development of Xinjiang Uygur Autonomous Region，Urumqi 830011，China

**Keywords:** 表面多孔颗粒, 全多孔颗粒, 手性固定相, 超临界流体色谱, 反相液相色谱, superficially porous particles, fully porous particles, chiral stationary phase, supercritical fluid chromatography （SFC）, reversed-phase liquid chromatography （RPLC）

## Abstract

使用紫外线和过氧化氢溶液去除失效色谱柱中硅胶填料表面的键合相，从而获得亚3 μm再生表面多孔颗粒（SPP）。将（2-羟乙基）-*β*-环糊精键合到再生SPP和3 μm商品化全多孔颗粒（FPP）表面，制备了基于亚3 μm SPP和3 μm FPP的手性固定相（SPP-CSP和FPP-CSP）。以外消旋佐匹克隆等10种分析物为手性分子探针，测试了两种CSP在超临界流体色谱（SFC）和反相液相色谱（RPLC）模式下的手性分离性能。结果表明，在相同的色谱模式及色谱条件下，与FPP-CSP相比，分析物对映体在SPP-CSP中的分离度更高，并且可以获得更尖锐的色谱峰形、更短的保留时间和更高的理论塔板数。当通过改变色谱条件获得相同分析时间时，FPP-CSP出现了显著的分离度损失，而SPP-CSP在提高流速以进一步缩短分析时间时，其分离度受到的负面影响较小，这为超快速手性分离提供了可能。SFC与RPLC表现出互补的手性识别范围，但在两者共同有效的分离范围内，SFC产生了更高的对映体分离度和更好的色谱性能。此外，流动相组成（尤其是添加剂种类）以及分析物自身的立体结构，都会影响对映体的分离效果。与相同粒径和颗粒结构的商品化（2-羟丙基）-*β*-环糊精（Hp-*β*-CD）手性色谱柱相比，所制备的SPP-CSP表现出独特的选择性，且该选择性随着色谱模式的改变而改变。然而，在被考察的两种色谱模式下，与商品化Hp-*β*-CD手性色谱柱相比，所制备SPP-CSP中分析物的色谱峰对称性较差，理论塔板数也较低。

## Abstract (en)

Chromatography remains a cornerstone technique in analyzing chiral compounds. The development and selection of chiral stationary phases （CSPs） are central to achieving high-resolution enantiomeric separation. This study presents a novel regeneration and functionalization strategy for the preparation of high-performance superficially porous particle （SPP）-CSP， offering a promising alternative to conventional fully porous particle （FPP）-CSP. First， bonded phases on the surface of spent chromatographic column silica packing materials were removed using ultraviolet light （*λ*=254 nm） and hydrogen peroxide solution （3%， mass fraction）， thereby obtaining sub-3 μm regenerated SPP. The regenerated SPP were then amino-functionalized. Subsequently， under mild reaction conditions， carbonyldiimidazole-activated （2-hydroxyethyl）-*β*-cyclodextrin （HE-*β*-CD） was bonded onto the surface of the amino-functionalized SPP to prepare the SPP-CSP. For comparative evaluation， a CSP based on 3 μm commercial FPPs was prepared. Additionally， a commercial （2-hydroxypropyl）-*β*-cyclodextrin （Hp-*β*-CD） chiral column with identical particle size and structure was selected as a reference. This commercial column was used to investigate the selectivity differences among different cyclodextrin derivatives. The enantioseparation and overall chromatographic performance of the CSPs were systematically evaluated under both supercritical fluid chromatography （SFC） and reversed-phase liquid chromatography （RPLC） modes. The evaluation was performed using ten chiral molecular probes： zopiclone， equol， 2-phenylpropionic acid， dobutamine， flutriafol， miconazole， folic acid， catechin， valacyclovir， and chlorphenamine. Results revealed that， under identical chromatographic conditions， the SPP-CSP consistently outperformed the FPP-CSP in terms of enantiomeric resolution， peak sharpness， theoretical plate number， and retention time efficiency. More importantly， the FPP-CSP exhibited a significant loss in resolution when chromatographic conditions were altered to yield equivalent analysis times. In contrast， the SPP-CSP showed only minor adverse effects on resolution when the flow rate was increased to shorten analysis time， highlighting its inherent suitability for ultrafast separations. Moreover， SFC and RPLC modes displayed complementary chiral recognition ranges. Within the overlapping separation scope of both modes， SFC mode achieved higher enantiomeric resolution and superior chromatographic performance. Notably， under RPLC mode， catechin showed high enantiomeric resolution on both CSPs， underscoring system-dependent selectivity differences. Multiple factors—including mobile phase composition （particularly the additive type）， and the stereochemical structure of the analytes—were found to significantly influence enantioseparation outcomes. Under SFC mode， none of the analytes could be eluted when acetonitrile was used as the mobile phase modifier， whereas alcohols effectively enabled elution， with weaker hydrophobicity correlating to stronger elution strength. Among the alcohols tested， methanol offered distinct advantages over isopropanol by maintaining lower backpressure and superior chromatographic performance without compromising resolution. Under RPLC mode， although isopropanol as a mobile phase component provided higher enantiomeric resolution， it resulted in relatively poorer chromatographic performance and higher column pressure drop. Using acetonitrile offered limited flexibility in adjusting elution strength. In both chromatographic modes， the influence trends of mobile phase additives on enantioseparation performance and overall column performance were generally consistent. Mobile phases with acidic additives were more suitable for analyzing acidic analytes such as 2-phenylpropionic acid and folic acid. Mobile phases with ammonium formate were better suited for analyzing basic analytes like chlorphenamine and flutriafol， as well as analytes with p*K*_a_ values near 7， such as zopiclone. In contrast， NH_3_ as an additive yielded unsatisfactory results. Compared with the commercial Hp-*β*-CD chiral column of the same particle size and structure， the SPP-CSP exhibited different selectivity， which varied with the chromatographic mode. Under RPLC mode， the SPP-CSP showed higher resolution for zopiclone， dobutamine， and folic acid. Under SFC mode， it demonstrated higher resolution for equol， folic acid， and valacyclovir. Despite these advances， the study has certain limitations. To enhance compatibility with mass spectrometric detection， normal-phase mode was not investigated. Additives commonly used in chiral compound analysis， such as triethylamine and ammonium trifluoroacetate， were also not employed. Polar organic solvent mode was not investigated either. Additionally， peak symmetry and theoretical plate numbers on the SPP-CSP were generally inferior to those observed on the commercial Hp-*β*-CD chiral column across both chromatographic modes.

20世纪60年代的“沙利度胺事件”从根本上改变了人们对化学手性影响人类健康的认识。据不完全统计，早在21世纪初期，中国、日本、欧洲等国家和地区药典收载的手性药物占比就已经达到40%~60%^［1，2］^。这些药物往往以外消旋体形式使用，一旦其手性遭到忽视，极易造成无法挽回的后果。色谱法因其形式多样、简便快速且成本低廉，被公认为手性化合物对映体分析的首选方法^［3-6］^。其中，手性固定相（chiral stationary phase，CSP）的选择是实现对映体高效分离的关键。随着制备技术的不断发展，大环糖肽^［7，8］^、多糖及其衍生物^［9，10］^、血清蛋白^［11，12］^、金属有机框架材料^［13，14］^、多孔有机笼材料^［15，16］^等相继被应用于手性色谱分析，分离对象也从最初的药物逐渐扩展至天然产物^［17，18］^、氨基酸^［19］^、环境污染物^［20，21］^等多个领域。环糊精（cyclodextrin，CD）及其衍生物作为第二代超分子化合物，以其独特的内疏水外亲水的空腔结构，易于与客体分子形成稳定包合物，兼具良好的手性识别能力与易得性，已被广泛应用于色谱固定相制备和流动相添加剂^［22-24］^。

根据Van Deemter方程和Giddings方程可知，色谱系统中的涡流扩散和传质阻力与填料粒径呈正相关。因此，在表面多孔颗粒（superficially porous particle， SPP）问世之前，减小填料颗粒粒径一直是提高色谱柱效率的主要途径。然而，更小的颗粒粒径不仅意味着更高的色谱柱压降（Δ*P*，即色谱柱入口和出口的压力差），流动相与填料之间产生的高摩擦热还会导致径向温度梯度和色谱峰展宽，这些因素使进一步提高色谱柱效率面临挑战。Kirkland教授等开发的5 μm及亚3 μm SPP，揭示了这种颗粒的巨大开发潜力^［25，26］^。由于构造特殊，SPP的粗糙度限制了填充柱床过程中气动装置释放高压浆料后颗粒的滑动，从而减少了径向床的不均匀性^［27］^。因此，在相同的色谱条件下，采用亚3 μm SPP色谱柱不仅能获得与亚2 μm全多孔颗粒（fully porous particle， FPP）色谱柱相近的分离效果，其色谱柱压降也接近于填充3 μm FPP的色谱柱^［25，27］^。此外，一些研究表明，SPP在多种色谱模式下均表现出优于FPP的手性分离性能^［28，29］^。

尽管SPP在色谱分离中具有显著优势，但其单分散制备技术门槛较高，商品化材料的价格亦十分昂贵，从技术上和经济上制约了相关应用研究的广泛开展。本文首先采用湿氧化法再生亚3 μm单分散SPP，随后在较为温和的反应条件下，将（2-羟乙基）-*β*-环糊精（HE-*β*-CD）键合到再生SPP和3 μm商品化FPP表面，制得两种CSP。在超临界流体色谱（supercritical fluid chromatography，SFC）和反相液相色谱（reversed-phase liquid chromatography，RPLC）两种模式下初步评估了这些CSP的手性分离性能。考察了流动相组成（尤其是添加剂种类）以及分析物自身立体结构对对映体分离效果的影响。此外，本文还引入具有相同粒径和颗粒结构的商品化（2-羟丙基）-*β*-环糊精（Hp-*β*-CD）手性色谱柱作为对照，以考察不同CD衍生物在手性识别中的选择性差异。

## 1 实验部分

### 1.1 仪器、试剂与材料

UPCC SFC系统、H-Class RPLC系统和QDa Ⅱ MS系统（美国Waters公司，均由Empower工作站控制）。TENSOR 27红外光谱仪（IR，德国Bruker公司）；VARIO EL Ⅲ元素分析仪（EA，德国Elementar公司）；EVO MA 10扫描电镜（SEM，德国Zeiss公司）；Milli-Q超纯水系统（美国Millipore公司）。失效的液相色谱柱（C8，100 mm×3.0 mm，2.7 μm，SPP，多孔层厚度0.5 μm，比表面积130 m^2^/g，美国Agilent公司）。3 μm商品化FPP（比表面积350 m^2^/g，苏州纳微科技股份有限公司）。商品化Hp-*β*-CD手性色谱柱（100 mm×2.1 mm，2.7 μm，SPP，多孔层厚度0.5 μm，美国Agilent公司）。

佐匹克隆、叶酸、伐昔洛韦、氯苯那敏、多巴酚丁胺标准品（外消旋，纯度≥92%，中国食品药品检定研究院）；雌马酚、2-苯基丙酸、儿茶素标准品（外消旋，纯度≥97%，上海阿拉丁生化科技股份有限公司）；粉唑醇、咪康唑标准品（外消旋，纯度≥94%，德国Dr. Ehrenstorfer 公司）。正庚烷、异丙醇、甲醇、乙腈、甲酸、甲酸铵（色谱纯，美国Thermo Fisher公司）。高纯CO_2_（纯度≥99.999%，北京氦普北分气体公司）。氨-甲醇溶液（2 mol/L，国药集团化学试剂北京有限公司）。30%（质量分数）过氧化氢（H_2_O_2_）溶液（优级纯，国药集团化学试剂陕西有限公司）。氨丙基三甲氧基硅烷、正丙胺、羰基二咪唑、HE-*β*-CD（上海阿拉丁生化科技股份有限公司）。

用甲醇配制1 mg/mL的单标储备液。移取各储备液适量，用正庚烷-甲醇（体积比为9∶1）溶液稀释成1 mg/L的混合标准使用液用于SFC分析，用甲醇-水（体积比为7∶3）溶液稀释成1 mg/L的混合标准使用液用于RPLC分析。

### 1.2 SPP-CSP和FPP-CSP的制备、表征和装填

参考Xiao等^［30］^去除介孔材料有机模板的方法再生SPP，并参考Jadhav等^［31］^快速合成CD键合硅胶的方法制备SPP-CSP和FPP-CSP。简述如下：使用H_2_O_2_溶液（质量分数为3%）和紫外线（*λ*=254 nm）去除失效色谱柱填料表面的键合相，获得亚3 μm再生SPP。氨基官能化再生SPP后，将HE-*β*-CD键合至再生SPP表面，得到SPP-CSP。制备路线如图1所示。SPP-CSP经清洗、干燥和表征后，制成SPP-CSP色谱柱（100 mm×3.0 mm）。对商品化FPP进行氨基官能化，并将HE-*β*-CD键合到其表面，制备相同色谱柱规格的FPP-CSP色谱柱作为参比。

**图1:**
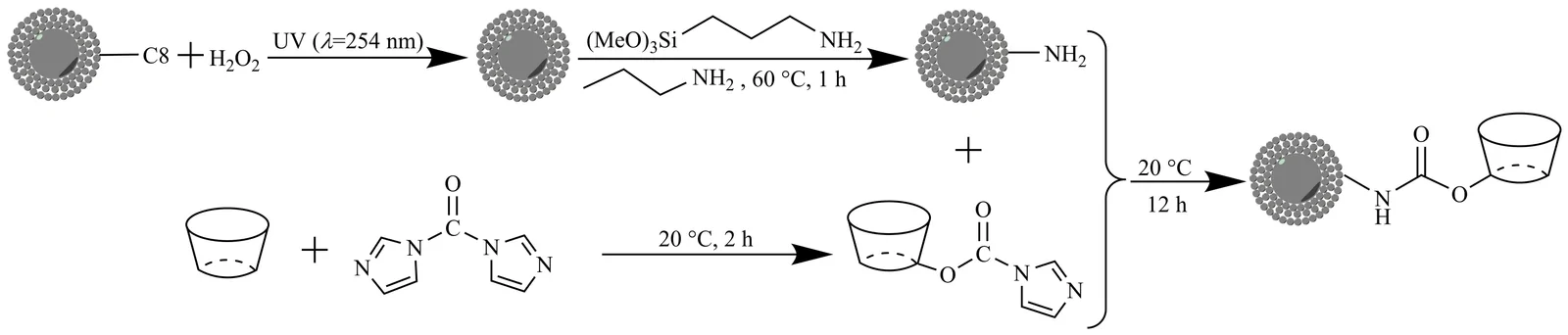
手性固定相的制备路线

分别采用IR和EA对制备的CSP进行表征。IR分析结果显示，与原始的再生SPP（图2a）相比，SPP-CSP的图谱（图2b）上新增了显著的特征吸收峰。其中，2 933 cm^-1^、2 862 cm^-1^处的吸收峰与C-H键的伸缩振动相关，1 654 cm^-1^处的吸收峰源自酰胺C=O键的伸缩振动，1 560 cm^-1^附近的吸收峰归属于N-H键的弯曲振动，1 420 cm^-1^附近的吸收峰源自C-N键的伸缩振动。以上结果表明，HE-*β*-CD被成功键合在SPP上，即SPP-CSP已成功制得。FPP-CSP（图2c）的红外吸收特征与SPP-CSP一致，说明FPP-CSP亦成功制得。

**图2:**
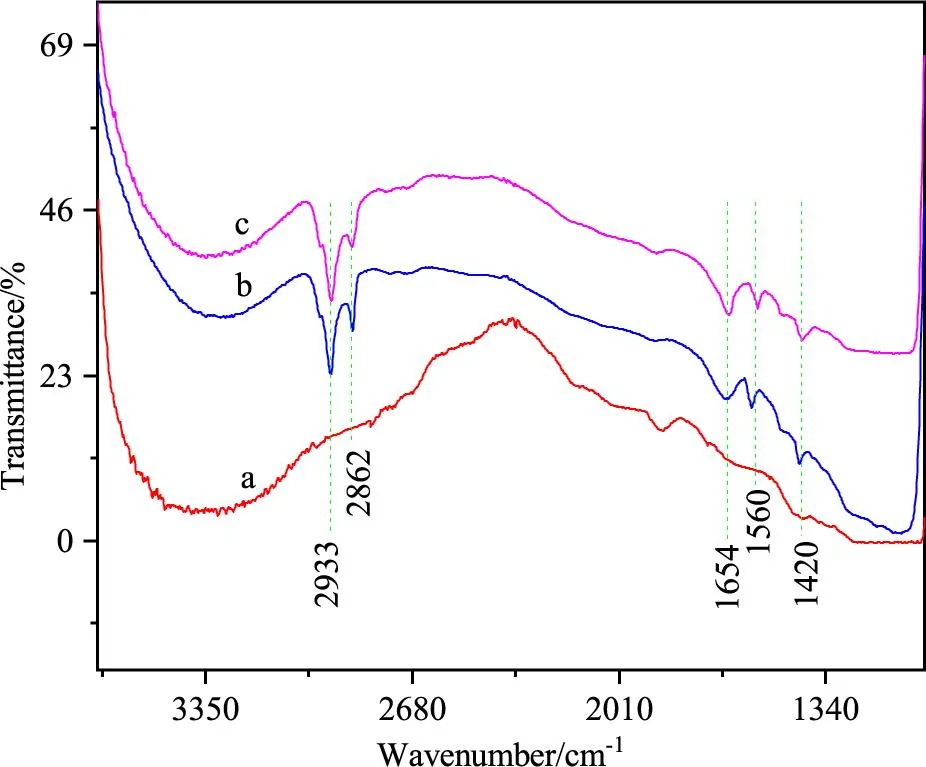
（a）再生SPP、（b）SPP-CSP和（c）FPP-CSP的红外光谱图

EA分析结果（表1）显示，与原始的再生SPP相比，CSP中碳元素、氮元素和氢元素的含量均显著增加，进一步证明了SPP-CSP和FPP-CSP的成功制备。根据Roy等^［28］^的方法，计算得出SPP-CSP和FPP-CSP的HE-*β*-CD键合量分别约为0.41 μmol/m^2^和0.40 μmol/m^2^。

**表1:** 再生SPP、SPP-CSP和FPP-CSP的元素分析结果

Material	Elemental contents/%		
	C	N	H
Regenerated SPP	0.11	0.12	0.60
SPP-CSP	3.73	0.25	0.86
FPP-CSP	9.55	0.31	0.92

### 1.3 实验条件

SFC条件 除另有说明外，流速1.5 mL/min，系统背压12.68 MPa，进样体积1.0 μL，柱温35 ℃。质谱补偿液为97%甲醇水溶液（含10 mmol/L甲酸铵），流速0.3 mL/min。主要流动相为CO_2_，分别以甲醇、乙腈、异丙醇以及含甲酸（体积分数为0.1%）、氨（1 mmol/L）和甲酸铵（25 mmol/L）的甲醇、乙腈、异丙醇为流动相改性剂，详见附表S1（www.chrom-China.com）。

RPLC条件 除另有说明外，流速1.0 mL/min，进样体积1.0 μL，柱温30 ℃。流动相分别为甲醇、乙腈、水以及含甲酸（体积分数为0.1%）、氨（1 mmol/L）和甲酸铵（25 mmol/L）的水溶液，详见附表S1。

MS条件 电喷雾电离源，正负离子同时扫描，扫描范围*m/z* 130~460，离子源温度150 ℃，去溶剂气流量1 000 L/h，去溶剂气温度500 ℃，锥孔电压15 V（正离子模式）、25 V（负离子模式），毛细管电压1.5 kV（正离子模式）、0.8 kV（负离子模式）。

## 2 结果与讨论

### 2.1 不同CSP的手性分离性能和色谱性能

以外消旋佐匹克隆等10种分析物为手性分子探针，考察了不同CSP在SFC和RPLC模式下的手性分离性能和整体色谱性能。所有分析物均在1.3节所述的流动相组成下进行了测试。为了便于比较，在相同色谱模式下使用不同CSP和不同流动相时，保持相同的流动相比例和流速。获得的色谱数据中，用于表征CSP手性分离性能的对映体分离度（*R*_s_）、选择性（*α*）以及用于表征色谱性能的第一个被洗脱对映体色谱峰的对称因子（*S*）、半高峰宽（*W*_h/2_）、理论塔板数（*N*）、保留时间（*t*_R_）等均由Empower软件给出。其中，各分析物对映体的分离度如图3所示，在最优流动相组成下的相关表征数据见附表S1。

**图3:**
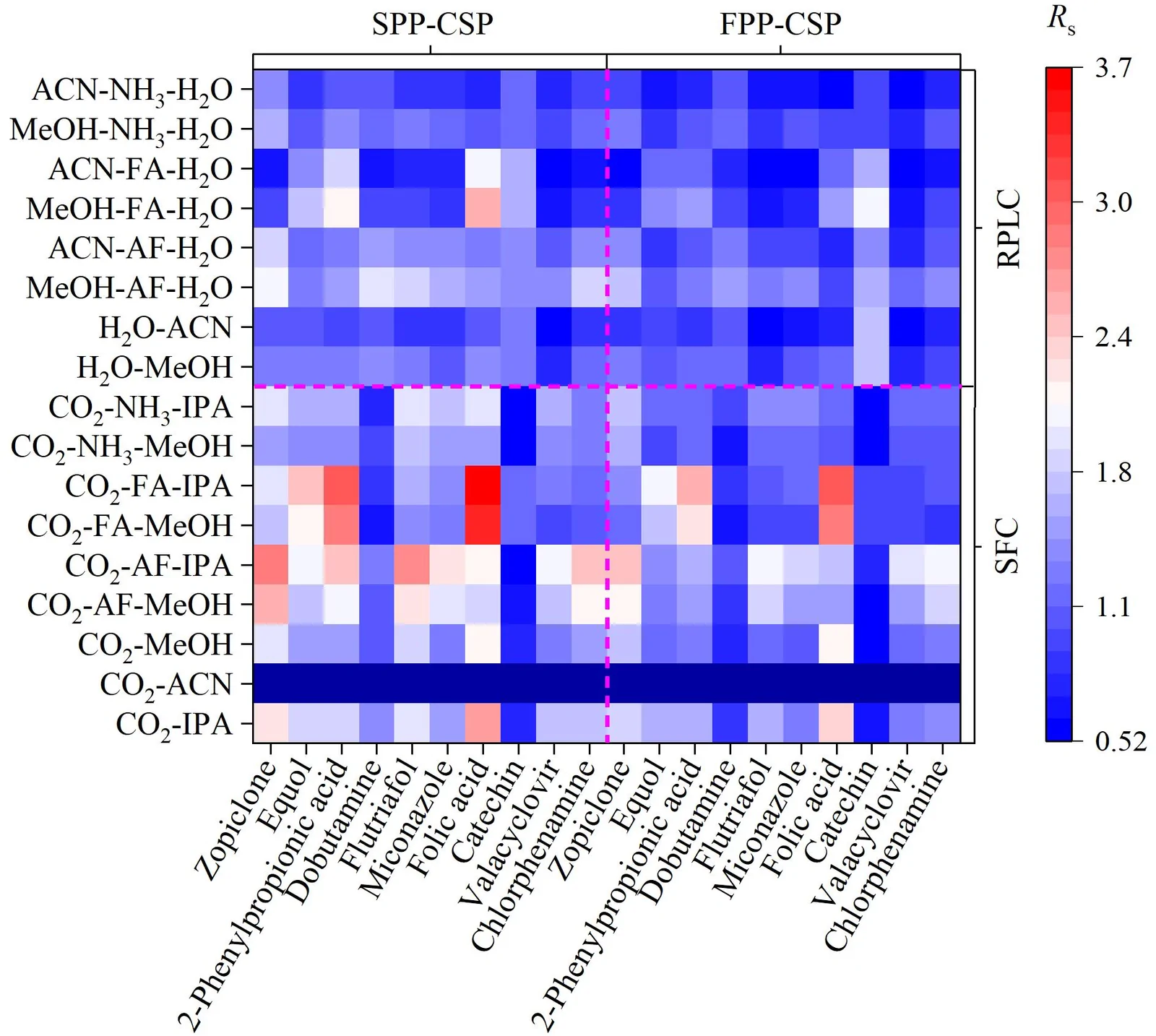
使用不同色谱模式、不同CSP和不同流动相时各分析物对映体的分离度

#### 2.1.1 色谱填料类型对对映体分离的影响

图3和图4a的结果表明，无论在SFC还是RPLC模式下，当流动相的比例和流速相同时，SPP-CSP展现出与FPP-CSP相当甚至略高的选择性和对映体分离度，并产生了更为尖锐的色谱峰形、更短的保留时间和更高的理论塔板数。虽然SPP-CSP的载碳量明显低于FPP-CSP，但二者的单位面积CD键合量几乎一样，这会降低SPP-CSP的样品负载量，但不会导致显著的选择性差异。当提高流动相的流速或流动相中有机溶剂的比例，使分析物在FPP-CSP上的色谱分析时间与SPP-CSP相同或接近时，FPP-CSP在两种色谱模式下都出现了显著的分离度损失（如图4b所示）。

**图4:**
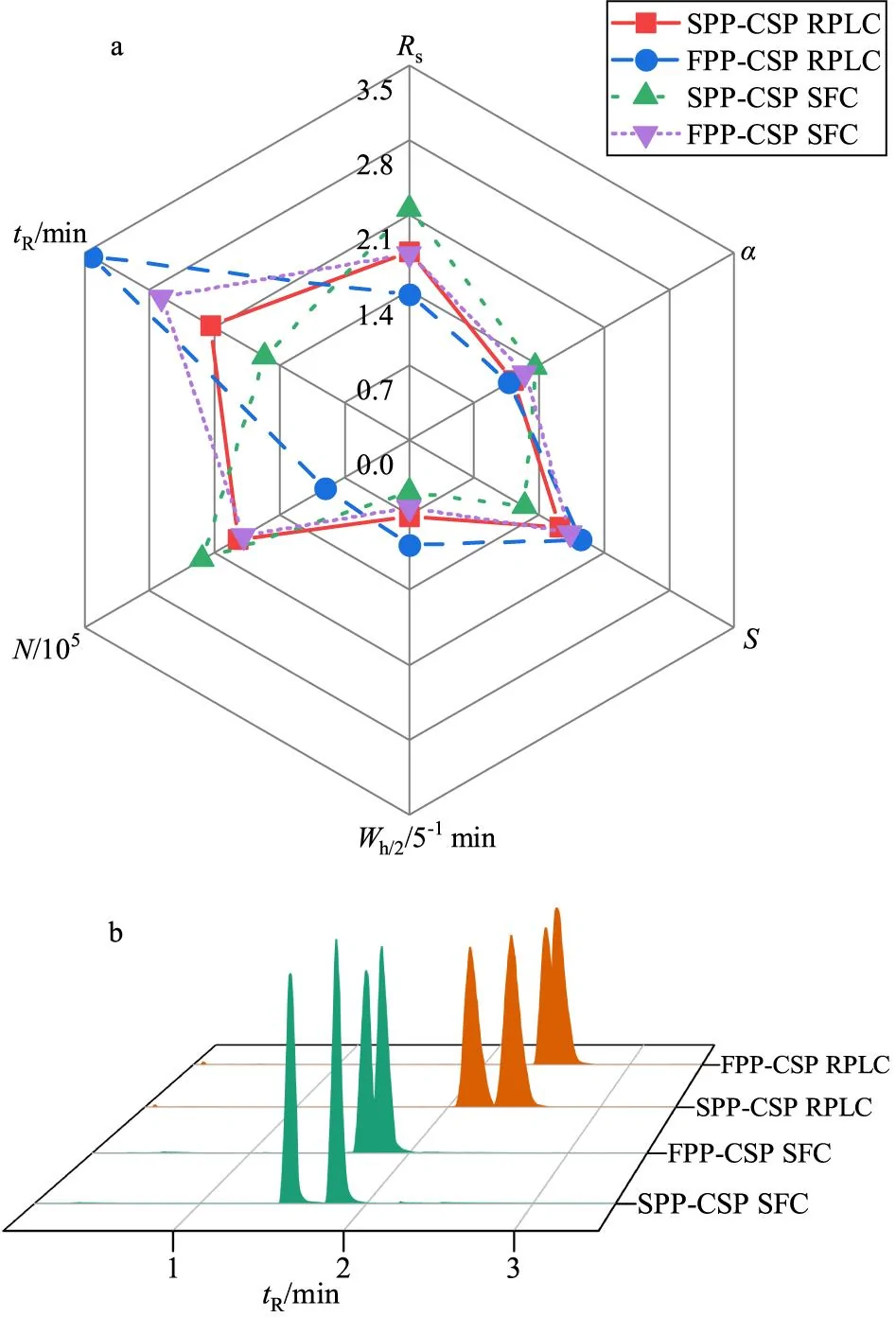
雌马酚对映体在（a）相同的色谱条件和（b）保持相同的色谱分析时间时的分离情况

当通过增加流动相流速以进一步缩短分析物的分析时间时，发现在SPP-CSP上增加流速不会像在FPP-CSP上那样对分离度产生显著的负面影响（如图5所示）。这一现象与Van Deemter理论高度吻合，该理论指出SPP-CSP具有更平坦的曲线特征，使其在较高流速下能更好地保持分离效率。此外，除增加RPLC有机溶剂的比例外，其他色谱参数的改变，如提高流动相的流速或增加SFC有机溶剂的比例等，均导致色谱柱压降出现不同程度的增加。综上所述，SPP-CSP在手性化合物对映体分离方面具有显著的潜在优势，这对超快速手性分离的研究具有重要意义。

**图5:**
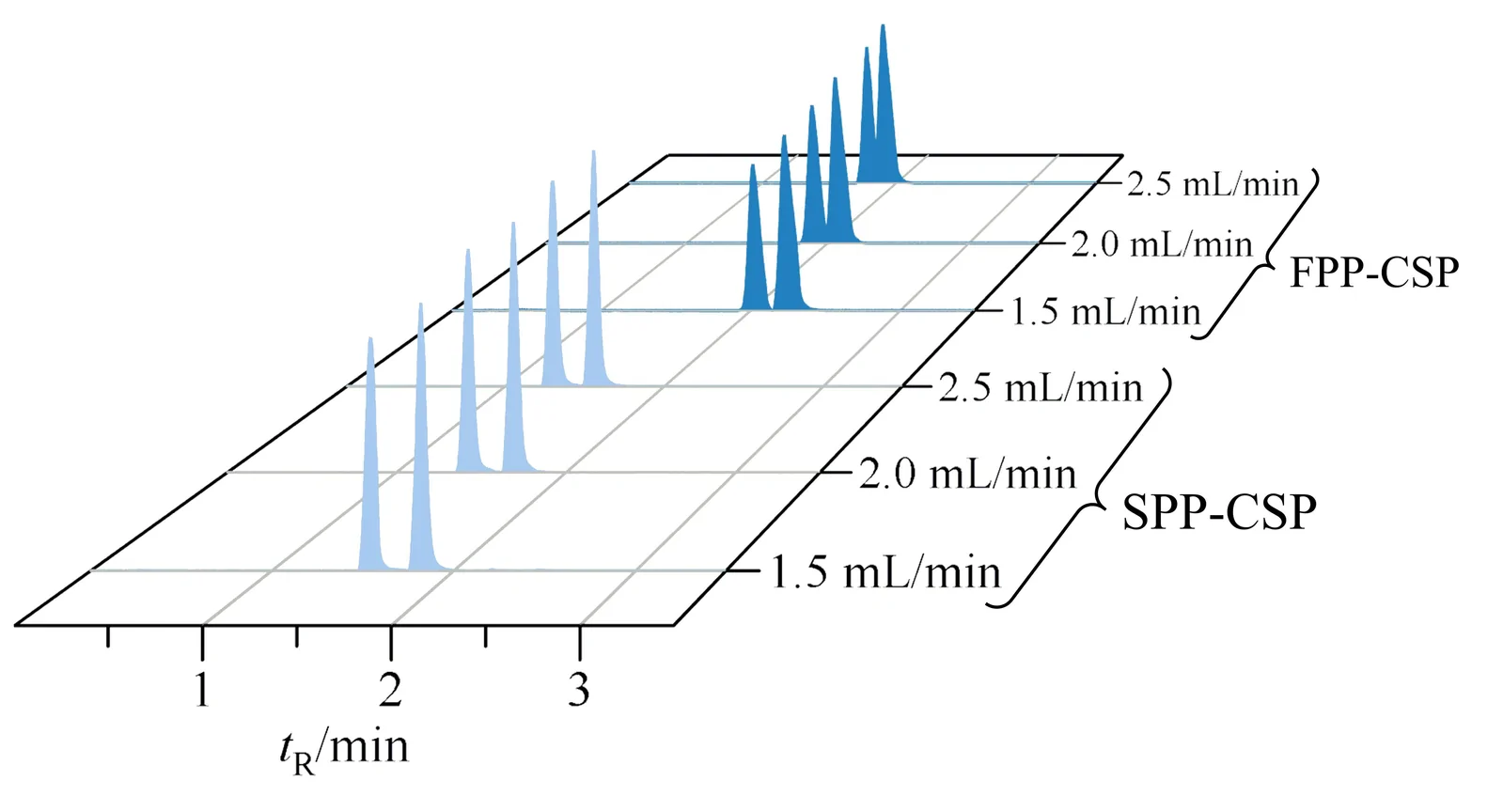
雌马酚对映体在不同流动相流速时的SFC-MS总离子流色谱图

#### 2.1.2 色谱模式对对映体分离的影响

图3和图4的结果表明，当色谱填料类型相同时，大多数分析物在SFC模式下可获得更高的对映体分离度，色谱峰形也更为尖锐，即使洗脱液处于亚临界状态，分离效果也没有明显变化，这与Roy等^［28］^观察到的结果基本一致。这是由于在SFC模式下，流动相的极性较低，强化了CD的疏水作用和包合作用，使分析物分子的非极性部分更容易嵌入其空腔，从而形成更为稳定的包合物。氢键作用等极性相互作用的影响也不容忽视。在SFC模式下，CD端口羟基与溶质间的氢键不会受到水的影响，能够协同发挥氢键作用和包合作用，因此具有更好的对映选择性。此外，在弱极性环境中，溶质的分子内氢键更为牢固、分子刚性更强，导致包合复合物形成的能量差异更大，也有利于增强对映选择性^［32］^。在RPLC中，流动相中的水会削弱甚至破坏氢键作用等极性相互作用，并降低包合作用的疏水驱动力，干扰溶质分子进入CD的空腔。

然而，在SFC模式下，过于强烈的氢键作用对对映体分离的影响并不总是积极的。对于含有丰富酚羟基的化合物（如儿茶素），强烈的分子内氢键导致其构象缺乏柔性，难以适配CD空腔，这种立体匹配失效削弱了CD的手性识别能力，使其在SFC模式下的分离度（*R*_s_=1.17）低于RPLC（*R*_s_=1.67）。此外，多巴酚丁胺在SFC模式下的对映体分离度（*R*_s_=0.84）也低于RPLC（*R*_s_=1.52）。这可能是因为多巴酚丁胺的手性中心远离其疏水环状结构，在SFC的弱极性环境中，分子倾向于形成折叠构象，导致手性中心被遮蔽。相比之下，RPLC的强极性环境破坏了多巴酚丁胺的分子内氢键，使手性中心充分暴露，实现了更有效的分离。此外，在这项工作中发现，在所有分析物中，分子越呈现线性，或者手性中心是环状结构的一部分，则越容易实现分离。

总之，SFC和RPLC表现出互补的手性识别范围，但在两种方法共同有效的分离范围内，SFC模式产生了更高的对映体分离度和更好的色谱性能。此外，由于超临界CO_2_具有低黏度、低表面张力、高传质速率等特性，使SFC模式的系统平衡时间更短，大幅提高了分析效率。

#### 2.1.3 流动相组成对对映体分离的影响

分析结果显示，在SFC模式下，使用甲醇、异丙醇和乙腈作为流动相改性剂对对映体分离的影响存在显著差异（如图3和图6所示）。当使用乙腈作为流动相改性剂时，各分析物均无法被洗脱。相比之下，当分别使用甲醇或异丙醇作为流动相改性剂时，所有分析物均能被洗脱。这是由于醇羟基能够与分析物分子竞争CSP上的氢键位点，因此醇类改性剂比乙腈更有利于分析物的洗脱，且疏水性越弱的醇从CSP氢键位点置换分析物的能力越强。进一步比较发现，使用甲醇作为流动相改性剂时各分析物的色谱峰形更为尖锐，保留时间更短，且色谱柱压降更低，尽管对映体分离度较低，但仍然令人满意（*R*_s_≥1.5）。综合考虑分离效率、色谱柱压降和操作便利性等因素，甲醇比异丙醇更适宜作为SFC模式的流动相改性剂。

**图6:**
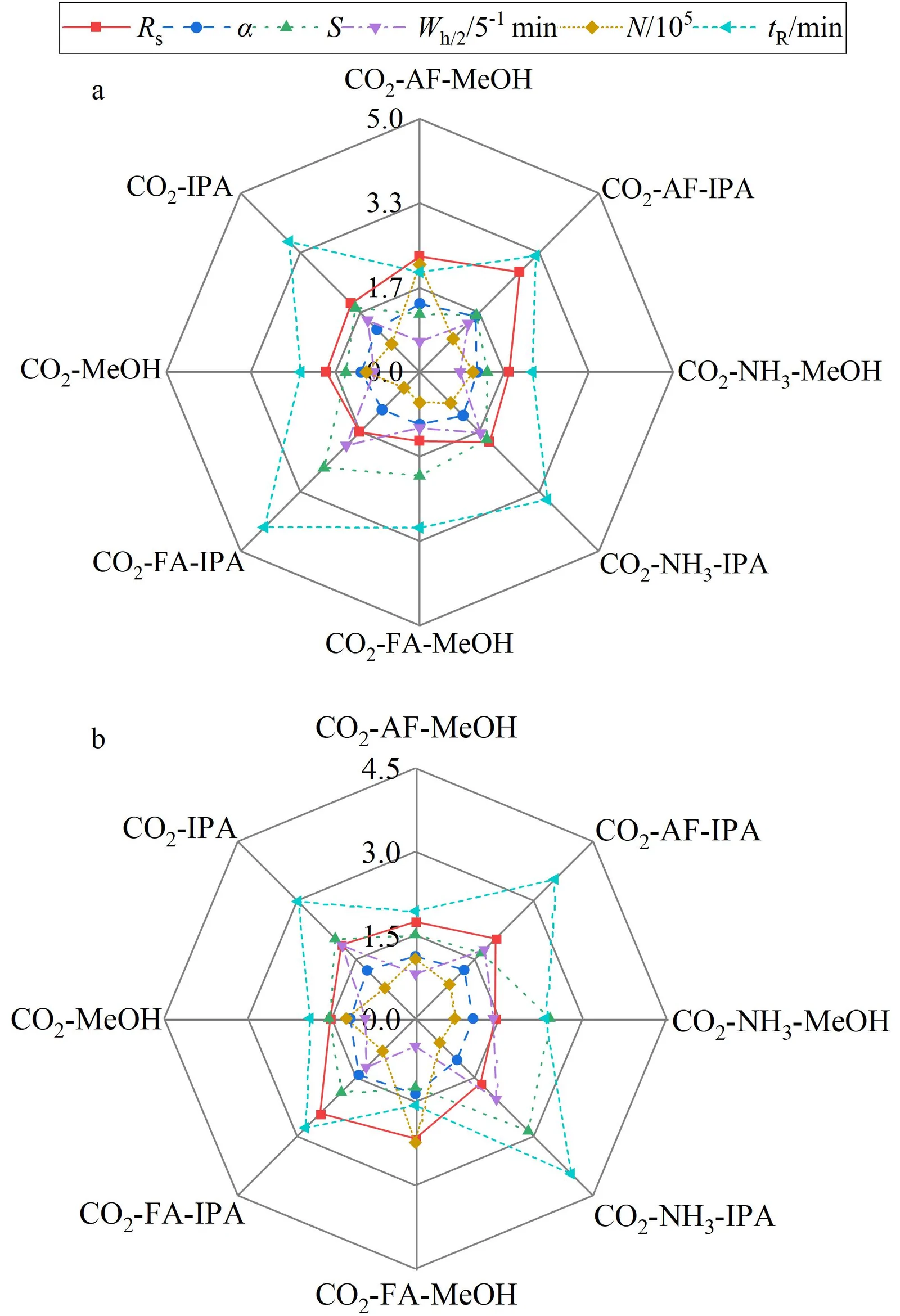
使用SPP-CSP色谱柱时（a）粉唑醇对映体和（b）雌马酚对映体在SFC不同流动相中的分离度、选择性以及第一个被洗脱对映体的对称因子、半高峰宽、理论塔板数及保留时间

在RPLC模式下，有机溶剂的洗脱能力与SFC模式下观察到的结果截然相反。与甲醇-水或乙腈-水流动相相比，异丙醇-水产生的色谱柱压降更高，这不利于实现超快速手性分离。因此，异丙醇并非理想的流动相组分。尽管甲醇的洗脱能力弱于乙腈，但其较低的实验成本和毒性以及仍能保证的足够分离度（*R*_s_≥1.5），使其成为更为适宜的流动相选择。

流动相添加剂对不同分析物对映体分离的影响存在显著差异。流动相添加剂通常用来调节流动相的离子强度、酸碱度以及固定相硅醇基团、可电离键合配体和溶质的电离状态等，从而调节溶质的保留时间和固定相的选择性，并改善色谱峰形。图3和图6的结果表明，当使用甲酸作为流动相添加剂时，2-苯基丙酸等酸性分析物能够获得更好的对映体分离度。这是由于酸性环境抑制了酸性分析物的电离，使其官能团保持质子化状态，有利于与CD形成氢键，增强对映体的识别能力。同时，酸性环境也减小了溶质与硅醇羟基之间的次级作用，改善了色谱峰形。对于氯苯那敏等碱性分析物，理论上更适合使用含氨的溶液进行分析。然而，在两种色谱模式下均发现，最优添加剂并非氨，而是甲酸铵。氨会导致硅醇羟基和键合配体过度去质子化，改变CSP表面的极性环境，从而削弱氢键作用并引起拖尾。使用甲酸铵作为流动相添加剂时，不仅能获得更好的对映体分离度，CSP的色谱性能也更好。对此，町田理论提供了更为深入的解释。町田等^［33］^认为，碱性物质保留因子的变化归因于分析物和缓冲阳离子进入疏水腔的竞争以及分析物和反阴离子之间离子对的形成，尽管竞争机制在不同性质的化合物中占主导地位，但观察到的结果可能归因于这两种机制的综合作用。在甲酸铵体系中，铵离子可以抑制硅醇羟基和键合配体过度去质子化，而且甲酸根与分析物更倾向于结合成为离子对，从而削弱铵离子的竞争优势，因此可以获得更好的分离效果。

#### 2.1.4 色谱柱温度对对映体分离的影响

为了考察温度对对映体分离的影响，以5 ℃为递增梯度，在25~40 ℃的色谱柱温度范围内对分析物进行分析。结果（附表S2）显示，在此范围内，温度没有对对映体分离度产生显著影响。当色谱柱温度升高时，分析物对映体在SFC模式下的保留时间逐渐增加。这是由于温度升高引起流体密度降低，从而减弱了流动相的洗脱能力。此外，温度升高也会引发有机改性剂的热脱附，进一步增强分析物与CSP之间的相互作用。对映体的分离度和CSP的选择性随着色谱柱温度的升高略有下降。这一现象符合手性识别的放热过程原理，即升高色谱柱温度加剧了分子的热运动，破坏了氢键等手性识别相互作用，缩小了对映体之间分配系数的差异。在RPLC模式下观察到的结果与SFC有所不同。随着色谱柱温度的升高，分析物对映体的保留时间逐渐缩短。虽然升高色谱柱温度使柱效有所提高，但仍无法补偿选择性的损失，最终导致分离度略有下降。这表明该模式下的手性识别以焓变差异（ΔΔ*H*<0）为主。

在考察的温度范围内，两种洗脱模式下的对映体洗脱顺序均未发生反转。这表明在该温度范围内，焓变差异始终占据主导地位，即便对于含有丰富酚羟基、能形成多重氢键作用的分析物也是如此（如儿茶素）。

### 2.2 SPP-CSP色谱柱与商品化Hp-***β***-CD手性色谱柱的手性分离性能比较

关于使用HE-*β*-CD制备CSP的研究报道相对较少。HE-*β*-CD的特点是其较短的碳链赋予其增强的亲水性和极性，可能带来针对特定分析物对映体的意外选择性。例如，Nowak及其同事^［34］^ 在利用毛细管电泳法分离卡西酮衍生物对映体时，观察到HE-*β*-CD相比其他CD衍生物具有独特优势。为了考察不同CD衍生物的选择性差异，本文选用粒径和颗粒结构一致的商品化Hp-*β*-CD手性色谱柱作为对照，在第1.3节所述的色谱条件下，对10种分析物的手性分离性能进行了评估，结果见图7。

**图7:**
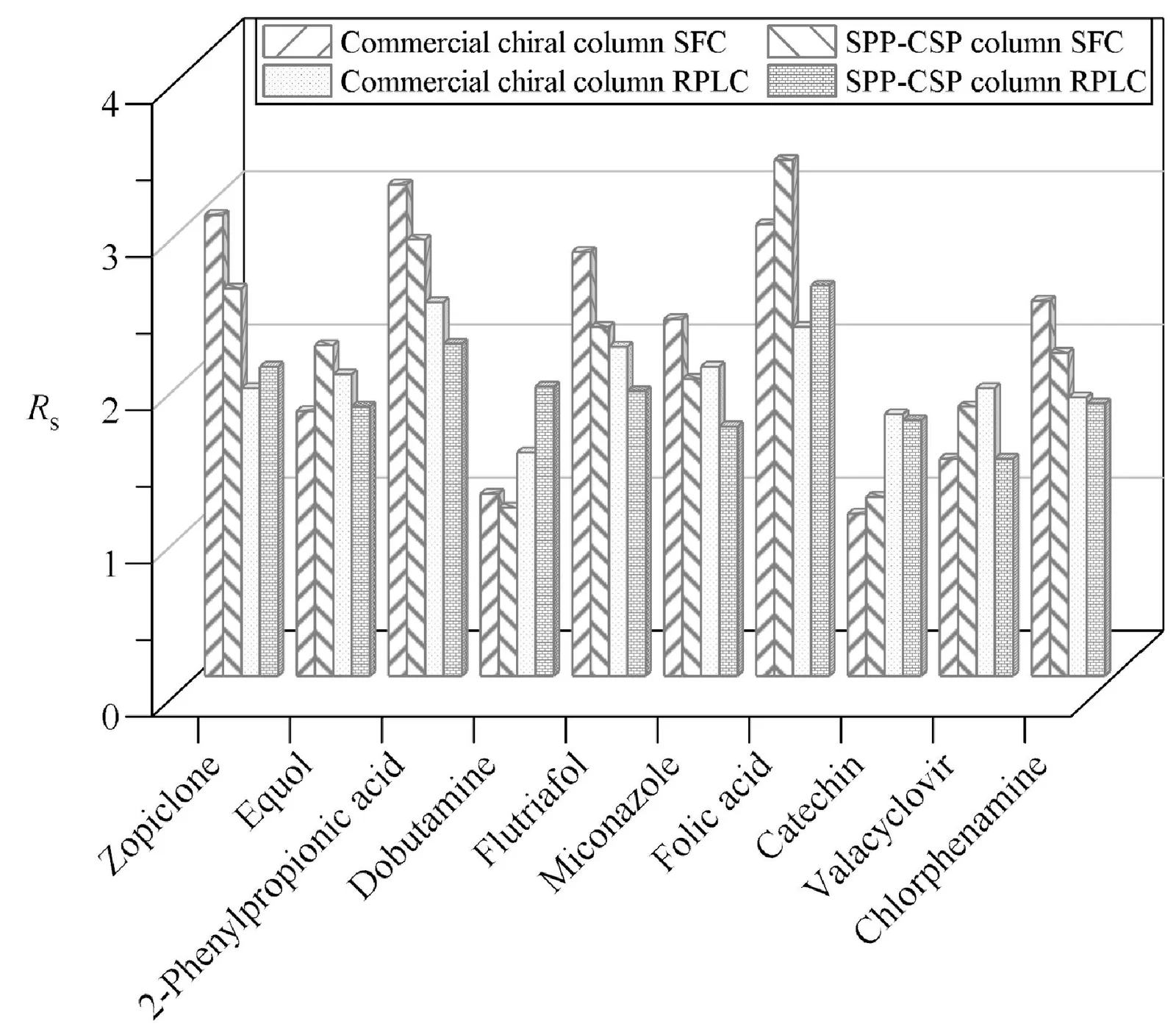
使用商品化Hp-***β***-CD手性色谱柱和SPP-CSP色谱柱进行分析时各分析物对映体的分离度

RPLC结果显示，SPP-CSP色谱柱在佐匹克隆、多巴酚丁胺和叶酸的对映体分离度方面具有更好的表现，而商品化Hp-*β*-CD手性色谱柱在雌马酚、2-苯基丙酸、粉唑醇、咪康唑和伐昔洛韦的对映体分离度方面表现更佳。对于儿茶素和氯苯那敏，两种色谱柱的手性分离性能无显著差异。这些结果很可能源于RPLC中手性识别机制的独特性：SPP-CSP色谱柱具有较高的亲水性和极性，除疏水及包合作用之外，其增强的极性相互作用使其更适合极性化合物的分析。相比之下，商品化Hp-*β*-CD手性色谱柱疏水性更强，尽管它也能参与极性相互作用，但更依赖疏水和包合相互作用。

由于SFC和RPLC流动相在极性上存在显著差异，两种CSP在SFC模式下的选择性也呈现明显不同。在SFC模式下，SPP-CSP色谱柱对雌马酚、叶酸和伐昔洛韦对映体的选择性和分离效果更好，而商品化Hp-*β*-CD手性色谱柱对2-苯基丙酸、佐匹克隆、粉唑醇、咪康唑和氯苯那敏的分离效果更佳。多巴酚丁胺和儿茶素的对映体分离度在此条件下无显著差异。

然而，在两种色谱模式下，分析物在所制备SPP-CSP色谱柱中的色谱峰对称性均低于商品化Hp-*β*-CD手性色谱柱，理论板数也较低。通过分析再生前后颗粒的SEM结果（附图S1）发现，再生后颗粒粒径没有发生改变，也没有出现破裂、塌陷等显著的外观变化。SPP-CSP色谱柱的色谱性能较差可能归因于其他多种因素，如键合量不足、键合不均匀或没有进行封端处理，导致硅醇羟基过度暴露形成活性位点，从而引起色谱峰展宽和拖尾，也可能是由于CSP 中存在金属杂质，或填充均匀性不佳、填充密度不足等。

## 3 结论

本文在较为温和的反应条件下，将再生SPP作为原料制备了手性分离材料。这一方法不仅减少了后处理过程中产生的污染，还显著降低了实验成本。随后，在不同色谱模式下，将其与近似粒径的FPP-CSP进行了手性分离性能和色谱性能比较，发现SPP-CSP在色谱分离对映异构体方面具有潜在优势，色谱性能也更好，且这些优势受到流动相流速变化的影响较小。这对于超快速手性分离的研究具有重要意义。此外，发现SFC和RPLC在可分离的手性化合物范围方面具有一定的互补性。在二者交叉的范围内，SFC展现出更高的分析效率和分离度。除不同的色谱模式外，手性化合物对映体的分离还受到流动相组成（尤其是添加剂种类）和化合物分子的立体结构等因素的影响。HE-*β*-CD键合SPP作为一种新型手性分析工具，在实现超快速分离方面展现出独特的优势和巨大的潜力。

然而，这项研究仍然存在一些不足。在被考察的两种色谱模式下，与商品化Hp-*β*-CD手性色谱柱相比，所制备SPP-CSP色谱柱的整体色谱性能较差。为了使流动相更适合质谱检测器分析，本文没有考察正相洗脱模式，也没有使用手性化合物分析中常用的三乙胺、三氟乙酸铵等流动相添加剂。此外，本文也没有考察极性有机溶剂模式。这些研究仍有待进一步开展。
